# Long-Term Effects of Integrated Strategies of Community Health Promotion on Diabetes Mellitus Mortality: a Natural Policy Experiment Based on Aggregated Longitudinal Secondary Data

**DOI:** 10.1007/s11524-021-00590-7

**Published:** 2021-11-19

**Authors:** Dominik Röding, Ulla Walter, Maren Dreier

**Affiliations:** Hannover Medical School, Institute for Epidemiology, Social Medicine and Health Systems Research, Carl-Neuberg Str. 1, 30625 Hannover, Germany

**Keywords:** Health in all policies, Intersectoral collaboration, Community-based prevention, Fixed effects, Natural experiment, Effectiveness

## Abstract

Integrated strategies of community health promotion (ISCHP) are based on intersectoral collaborations using the Health in All Policies approach to address determinants of health. While effects on health determinants have been shown, evidence on the effectiveness of ISCHP on health outcomes is scarce. The aim of this study is to assess the long-term effects of ISCHP on diabetes mellitus mortality (DMM) in German communities. A nonrandomized evaluation based on secondary county-level official data (1998–2016) was performed. In April 2019, 149 communities in Germany with ISCHP out of 401 were identified. Communities with < 5 measurements of DMM, starting before 1999 or after 2015, were excluded. Analyses included 65 communities with ISCHP (IG) and 124 without ISCHP (CG). ISCHP ran for a mean of 5.6 years. Fixed effects (FE) models were used to estimate effects of ISCHP and duration on DMM taking into account the time-varying average age. The FE estimator for DMM is *b* =  − 2.48 (95% CI − 3.45 to − 1.51) for IG vs. CG and b =  − 0.30 (95% CI − 0.46 to − 0.14) for ISCHP duration (0–16 years). In the first year of an ISCHP, a reduction of the annual DMM of 0.3 per 100,000 population (1%), and in the 16th year of 4.8 (14%) was achieved. This study provides preliminary evidence of the effectiveness of ISCHP in Germany. Limitations include inaccuracies to classify IG and CG and possible selection bias. Longitudinal county-level data may be an efficient data source to evaluate complex interventions, thereby contributing to further strengthen evidence-based integrated health promotion.

**Supplementary Information:**

The online version contains supplementary material available at 10.1007/s11524-021-00590-7.

## Background

Health in All Policies (HiAP) describes a holistic societal model in which policies are applied as interventions to promote the health of a population [1]. Interventions based on HiAP typically target at communities or settings and may address various health-related sectors [1]. The WHO mentioned the concept of HiAP in 2010 in a statement; however, numerous HiAP-like interventions have been implemented (long) before, such as the WHO Healthy Cities movement, showing that by targeting the population as a whole including the physical and social environment, even modest changes of determinants of health may have a large public health impact [2]. In line with this population-based strategy, research focused on developing, implementing, and evaluating comprehensive community prevention approaches (CCPA) based on socioecological models [3]. While meta-analyses and systematic reviews provide clear evidence on the effectiveness of CCPA on health behaviours, evidence on health consequences like morbidity or mortality remains inconsistent [4–7].

**Fig. 1:**
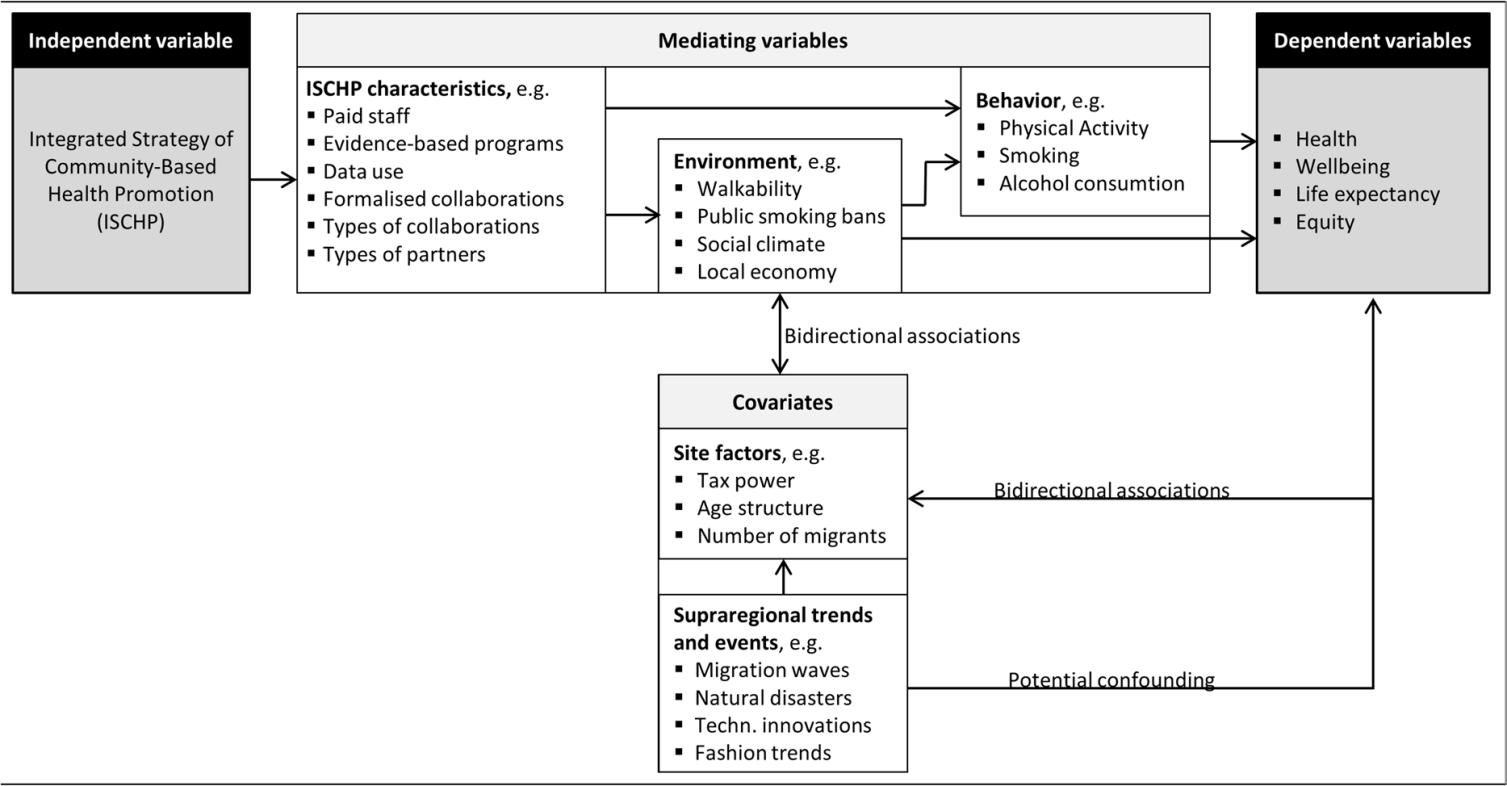
Logical model to visualize causal pathways of ISCHP-based effects

In Germany, communities have started to establish Integrated Strategies of Community Health Promotion (ISCHP), since the late 1980s with starting the German Healthy Cities Network [8]. ISCHP are CCPA based on the framework of health determinants and of Health in All Policies (HiAP) [1, 9]. Meanwhile, several initiatives at the state and federal levels support ISCHP [8]. The model in Fig. 1 shows possible causal pathways between the intervention ISCHP and the expected outcomes including important mediating and moderating parameters. Typically, ISCHP mediated by policy-induced changes in health determinants need a longer latency period to produce measurable effects on health outcomes. However, as ISCHP have so far evaluated only in a formative way despite a sufficient period of time, evidence on their effectiveness is still lacking [8, 10].

To examine retrospectively the effect of ISCHP on health, we classified the initiation of an ISCHP or not as a natural policy experiment as policies were not initiated or allocated by the researchers [13, 14]. We chose diabetes mortality as a relevant health outcome because we assumed that health outcomes related to diabetes mellitus are sensitive to ISCHP as healthy diet and promotion of physical activity are common (intermediate) targets of ISCHP [15, 16]. Further reasons include that diabetes is highly prevalent in Germany contributing to a substantial burden of disease and the data availability [17, 18]. We used fixed effects analysis, thereby taking advantage of the longitudinal data and of implicitly eliminating time-invariant (socioeconomic) confounders to enhance validity/causality. The two leading hypotheses of our study are (1) diabetes mellitus mortality (DMM) increases less/decreases more in communities, which initiated an ISCHP; (2) this effect is stronger the longer ISCHP runs.

## Methods

This evaluation follows the ‘Transparent Reporting of Evaluations with Nonrandomized Designs’ (TREND) statement [19]. Ethical approval was obtained from the Ethics Committee of Hannover Medical School (reference number 10052_BO_K_2021).

### Study Design, Assignment Method, and Eligibility 

We used a natural policy experiment to retrospectively evaluate the long-term effects of ISCHP on DMM in German communities (counties and independent towns) [20, 21]. The assignment method is self-selection from comparison group (communities without ISCHP since 1998) by default into the intervention group by initiating an ISCHP in the years 1999 to 2015. The units of analysis are communities. Due to the holistic approach of ISCHP, with the exception of non-modifiable variables like age, gender, genetics, ethnicity, and supra-regional events and trends, all determinants of health that community can influence do no more represent potential confounders rather than mediating variables (Fig. 1). Based on McDowell et al. [22], we understand ISCHP as a population determinant to be captured by a global measure and DMM as an individual outcome that can be captured by an aggregated measure. Our analyses include county-level time series data of yearly DMM between 1998 and 2016. Eligible were all communities with a minimum of 5 years data available on the number of persons who died of diabetes mellitus, an annual sequence was not required. Excluded were all communities that initiated an ISCHP before 1999 or after 2015.

### Data Sources and Data Linkage

Exposure (ISCHP as the self-selected intervention): We used databases, member registers, and grey literature [E-Supplement] to identify all German communities (independent towns, counties, towns, and rural communities) with an ISCHP (*n* = 214). The starting year was the year in which these communities first either participated in a program aimed at building an ISCHP or launched a project to build an ISCHP. We use the latter as a proxy measure for the year the communities initiated the ISCHP. Data collection took part in April 2019.

Outcome diabetes mellitus mortality (DMM): County-level data of the years 1998 to 2016 on population size (*n* = 489) and number of death of diabetes mellitus (*n* = 243) were downloaded from www.regionalstatistik.de in May 2019. County-level data of relevant time-varying confounders for the years from 1998 to 2016 on the mean age, proportion of foreigners, and proportion of women were downloaded from www.inkar.de in May 2019.

Data on exposure that not already referred to the county-level were manually aggregated to this level. Next, exposure data were manually merged with the data from www.inkar.de using the municipality names. As both the data from www.inkar.de and the data from www.regionalstatistik.de include the official municipality key, these data could be merged in an automated way using IBM SPSS Version 26.

### Intervention and Comparison Conditions

#### Communities with an ISCHP

ISCHP is described as an overall strategic and coordinated approach and the collaboration of various society and policy sectors with the aim of creating health-promoting living environments and services [23]. ISCHP address a range of determinants of health and a range of target groups with special attention to increasing the health opportunities for people in socioeconomic risk situations [23]. To this end, preventive services and measures are coordinated across municipal departmental boundaries and with the involvement of actors from outside the administration and the target groups [23]. Therefore ISCHP require a form of steering that is based on collaboration and consensus-building and coordinates both horizontally between different society and policy sectors and vertically between different levels of action [23]. A central role in steering played the municipal administration [23]. The German Cooperation Network ‘Equity in Health’ published five booklets about how to develop and maintain an ISCHP. There are further guidelines to implement an ISCHP [24, 25].

#### Communities without an ISCHP

It can be assumed that in Germany, even in communities without ISCHP, concepts and principles are used that belong to the core features of an ISCHP. However, the prevention strategy in communities without an ISCHP is certainly less holistic, addresses only a few target groups and health outcomes, and therefore focuses only on a subset of the social determinants of health. Assumingly, in communities without an ISCHP, intersectoral networking and collaboration is less intensive in most cases.

### Statistical Analysis

Linear fixed effects (FE) models were used to estimate the effects of the time-varying independent variables ISCHP initiation and of ISCHP duration on the time-varying dependent variable DMM [26]. FE models completely ignore the between-unit variation and use only within-unit variation to estimate the effects of time-varying independent variables on a time-varying dependent variable. Consequently, FE model estimates are completely controlled for all (un)observed time-invariant variables. Relevant time-varying variables have to be observed and to be considered in the model to get unbiased estimates [14, 26]. The FE models were conducted in two ways using IBM SPSS Version 26. (A) The FE models presented in tables were modelled using the least squares dummy variable (LSDV) approach [26]. Here, the units of analysis (communities) included in the model are coded as dummy variables. Since graphical and test-statistical analysis could not exclude heteroscedasticity, the FE models were computed with robust standard errors [27]. (B) The FE models presented in the grouped scatter plots are based on FE transformation that subtracts the unit-specific mean value (the mean of all time points) for each time-varying variable and performs an ordinary least squares (OLS) regression with these demeaned data [26, 28].

#### Dependent Variable

The time-varying health outcome measure is the crude annual diabetes mellitus mortality rate per 100,000 residents in the communities for the years 1998 to 2016. This rate was calculated using annually reported data on population size and persons who died from diabetes mellitus. For some analyses, we performed a FE transformation on this variable.

#### Independent Variables

The initiation of an ISCHP was coded as a time-variant dichotomous variable. For the year 1998, this variable was set to 0 for all communities. The value 0 indicates that the community does not have an ISCHP (comparison group). Communities that did not initiate an ISCHP between 1999 and 2015 have a value of 0 in this variable for all years. For communities that initiated an ISCHP between 1999 and 2015, this variable was then given a value of 1 from the year of initiation. The second outcome, the duration of the ISCHP, was coded as a time-variant quasi-metric (ordinal) variable. This variable was also set to the value 0 for all communities for the year 1998 that means there is no ISCHP running in the community. Communities that did not initiate an ISCHP from 1999 to 2015 have a value of 0 in this variable for all years. Communities that initiated an ISCHP during this period have a value of 1 in the year of initiation, and for subsequent years this value increased by 1 each year. It should be noted that this variable thus reflects the interaction effect of ISCHP initiation (0 = no ISCHP vs. 1 = ISCHP initiated) and ISCHP duration (range: 0–17 years), because the product of these two variables is equivalent of ISCHP duration.

#### Time-Variant Covariates

As a measure of the changing age structure, we used a time-varying variable on the community-specific average age. As measures of changing proportions of migrants and women, we used time-varying variables on the municipality-specific proportion of migrants and proportion of women. The time trend was coded as a time-varying quasi-metric (ordinal) variable. For the year 1998, the variable has the value 0 and for the following years, the value increases by 1 each year. Due to consistently missing values in 2011 for the dependent variable, the year 2011 was not included in the analyses.

At the beginning of the analyses, outliers were identified in the DMM data. This analysis shows that data points with a DMM > 75 per 100,000 represent outliers. Of the total 3402 data points in our dataset, 244 (7%) were identified as outliers and excluded. Excluded data were equally distributed in the IG and CG. Next, the change-in-estimate-criterion was used to analyse which of the time-varying covariates should be included as confounders in the FE models [29]. While including the average age into the model changes the effect estimate for ISCHP initiation by 322%, including the proportion of immigrants changes the effect estimate by only 3% and the proportion of women by 2%. Therefore, the FE models have been adjusted for average age only. In FE models, time-constant covariates (e.g. baseline values or geographic location) are always fully controlled for. As analysis on collinearities of the independent variables (ISCHP duration, time trend, and average age) detects collinearity between time trend and average age (*r* = 0.649; *p* < 0.001), only one of the two variables is included in the FE models.

## Results

**Fig. 2:**
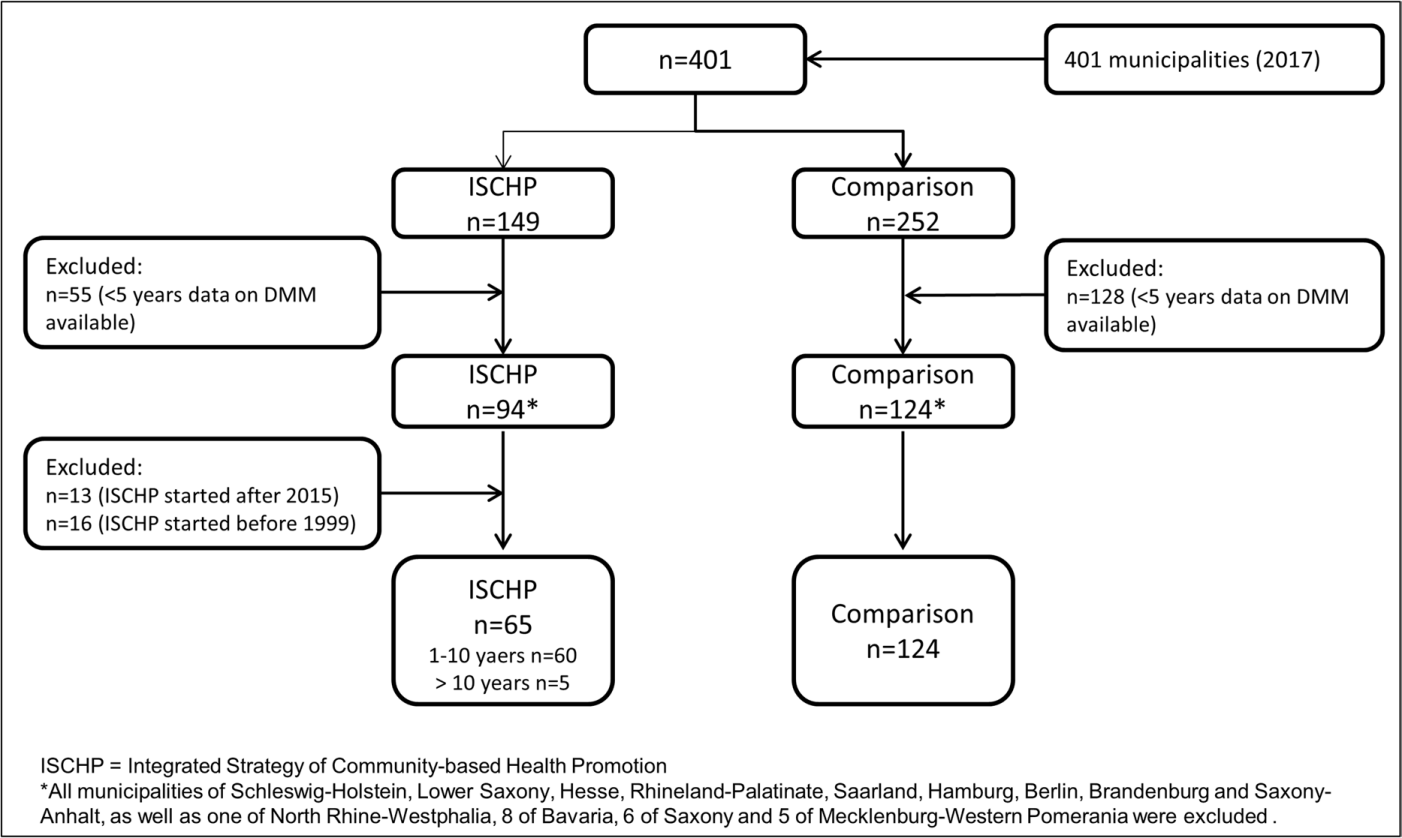
Flow diagram of the study population

Of all 401 German communities (counties and independent towns), we identified 149 who had initiated an ISCHP and 252 who had not (Fig. 2). Exclusion of communities with fewer than five data points for the outcome and of communities who initiated an ISCHP before 1999 or after 2015 resulted in an intervention group (IG) of 65 and a control group (CG) of 124 communities. The ISCHP duration ranged from 1 to 17 years with a mean duration of 5.6 years (median: 5 years). The mean number of annual data points for the outcome measurement is 17 (range: 7–18) out of possible 18 data points. In 1998, the mean DMM was 19.1 per 100,000 in the future IGs and 25.7 per 100,000 in the CG (Table 1). In 2007, the DMM was approximately 26 in both groups and increased to 28.0 in the IGs and to 38.5 per 100,000 in the CGs by 2016.

**Table 1:** Characteristics of the study population

	Intervention group (*n* = 65)		Comparison group (*n* = 124)		Germany (*n* = 401)	
	% / mean	SD	% / mean	SD	% / mean	SD
Region						
West Germany	81.0		63.4		69.3	
East Germany	19.0		36.6		30.7	
Type of county						
Independent town	43.7		16.6		25.6	
County	56.3		83.4		74.4	
First baseline in this multiple baseline design is 1998						
Population 1998	282,829	330,570	140,553	81,686	188,902	214,313
Female Proportion 1998	51.4	0.7	51.1	0.7	51.2	0.7
Birth per 1,000 Population 1998	9.6	1.5	9.5	1.7	9.6	1.7
Average Age 1998	40.2	1.3	40.1	1.4	40.1	1.4
Migrant Proportion 1998	9.4	5.2	6.2	4.2	7.4	4.8
Unemployment rate 1998	11.0	4.0	10.6	4.8	10.7	4.5
Highest school qualification 1998	23.4	6.9	20.9	7.5	21.8	7.4
Tax power per capita 1998	500 €	161 €	429 €	157 €	455 €	162 €
Age standardised mortality 1998 per 100,000	1,112.2	362.4	984.0	439.7	1,027.8	419.0
Diabetes mortality 1998 per 100,000	19.1	10,9	25.7	14.2	22.9	13.2

The communities of the IG are more often located in West Germany and include more often independent towns compared to the CG (Table 1). The mean population size of the IG is larger compared to the CG. The mean proportion of women, the mean birth rate, and the mean age are equally distributed in both groups. The mean proportion of migrants, of the highest school qualification, the mean unemployment rate, the mean tax power, and the age-standardized mortality are slightly higher in the IG compared to the CG.

**Fig. 3:**
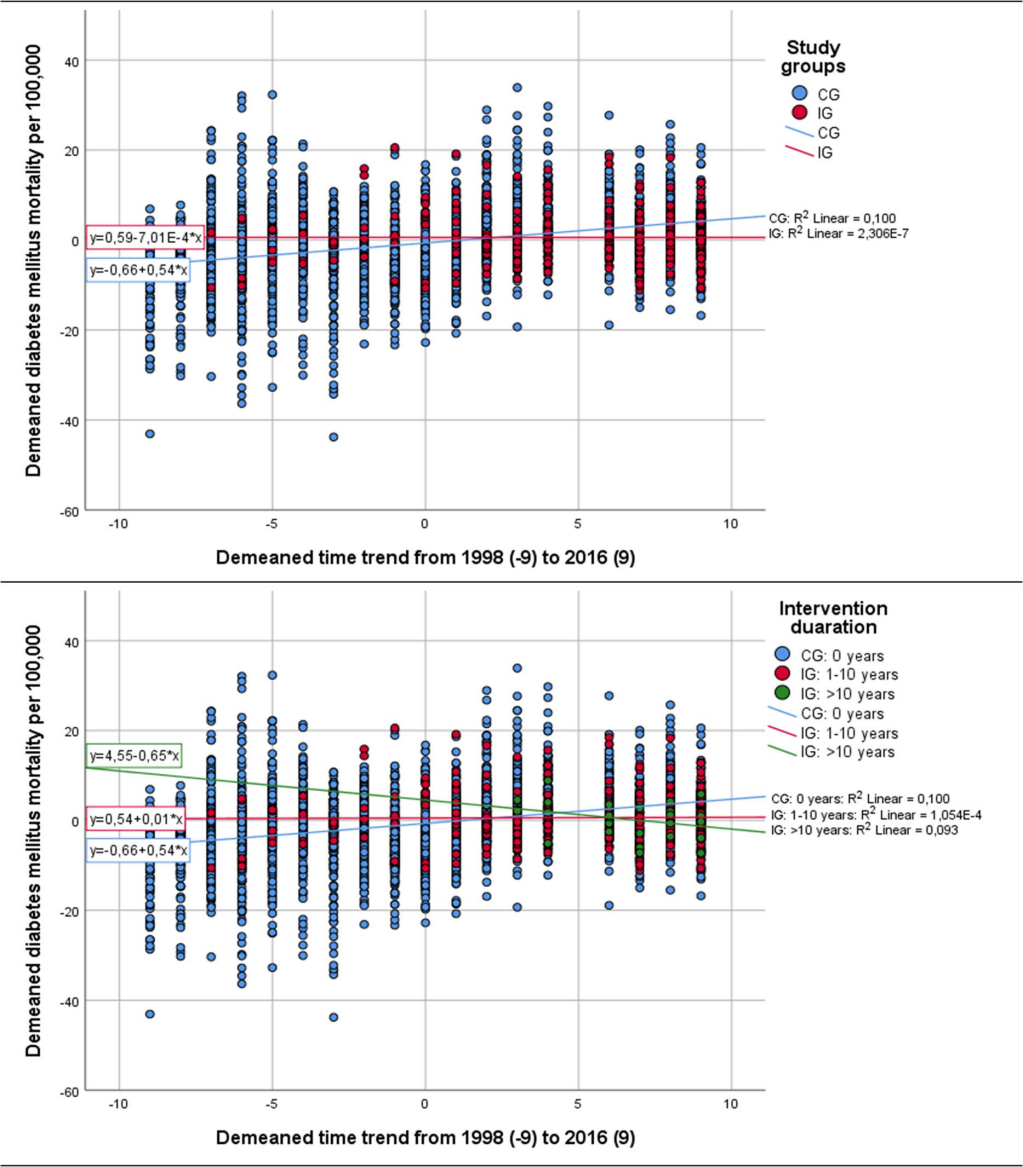
Starting/duration of ISCHP and trend in annual diabetes mortality over time. Legend: Grouped scatter plot of the demeaned DMM (each data point represents the deviation of the mean DMM of one IG or CG from its own mean DMM over time) during 1998–2016. OLS regression analysis using these demeaned DMM qualifies as a FE regression. Groups are IG (*n* = 65; observations = 334) and CG (*n* = 124; observations = 2824), respectively, groups are IG with ISCHP of 1–10 years (*n* = 60; observations 308), IG with ISCHP of more than 10 years duration (*n* = 5; observations 308), and CG without ISCHP (*n* = 124; observations 2,824)

Figure 3 shows that communities having initiated ISCHP experienced a nearly stable DMM (increase of 0.0007 per 100,000 and year), while communities of the CG developed an increase in DMM of 0.54 per 100,000 and year. The DMM in communities with more than 10 years of ISCHP duration tended to decrease (Fig. 3).

In communities with ISCHP, FE model analysis found a mean annual decline in DMM of 2.5 per 100,000 persons compared to the CG (Table 2). A higher average age was associated with an increased DMM. The duration of ISCHP was negatively associated with the DMM (Table 2). Each year with ISCHP, the DMM reduces on average by 0.3 per 100,000 persons, decreasing to an annually DMM by 4.8 per 100,000 persons after 16 years of ISCHP. Analyses included ISCHP with a mean duration of 4.4 years ranging from 1 to 16. A higher average age was associated with a higher DMM.

**Table 2:** Two FE regression models analysing the effect of (a) starting an ISCHP and (b) its duration on the annual diabetes mortality

	Regression coefficient b	Robust standard error	95% confidence interval		*P* value
Starting an ISCHP					
Constant term	− 17.528	6.746	− 30.756 -	− 4.301	0.009
Starting an ISCHP	− 2.479	0.494	− 3.447 -	− 1.510	< 0.001
Average age	1.817	0.099	1.623 -	2.010	< 0.001
Duration of an ISCHP					
Constant term	− 4.301	6.670	− 27.380 -	− 1.222	0.032
ISCHP duration (0–17 years)	− 0.298	0.083	− 0.461 -	− 0.136	< 0.001
Average age	1.744	0.096	1.555 -	1.933	< 0.001

## Discussion

We retrospectively analysed the effect of ISCHP started between 1999 and 2015 on diabetes mortality using a natural experiment based on aggregated longitudinal secondary data on county-level. From all 401 German communities, we included 65 as IG und 125 as CG. The FE models showed that ISCHP are associated with an average absolute reduction of DMM of 2.5 per 100,000 persons each year they run. A longer duration was associated with higher effects. To our knowledge, this is the first summative evaluation of ISCHP in Germany.

Applying advanced methods based on the latest research knowledge to provide evidence on the effects of complex health promotion programs is one of the major strengths of our study. There is a broad consensus that natural experiments and quasi-experimental study designs are appropriate for evaluating the effectiveness of CCPA [13, 14, 20, 30]. This is especially true when the focus is on population-level effects. The FE analysis we used is one out of six statistical analysis methods recommended to validly analyse data of non-randomized studies [14]. A major advantage of FE analyses is that they lead to conservative parameter estimates being close to the true value [14]. Other studies have also shown that time series analysis of small-area-level data is appropriate to demonstrate long-term effects of complex public health interventions [31–33].

Compared to randomized studies, natural experiments or quasi-experimental studies are prone to baseline imbalances. In this study, we cannot exclude a selection bias as the communities had chosen whether to start ISCHP or not and the reason for their decision remained unknown. Motivation to implement ISCHP could either include concerns on community health as well as to further promote health in already health-conscious, well-being communities. Comparing the available socio-demographic indicators in 1998 between IG and CG, no consistent result emerges to the question whether one study group might be more privileged than the other one. However, time constant confounders are automatically adjusted for in FE models.

Our results are in line with two other long-term community-based prevention programs. The Stockholm Diabetes Prevention Program cumulatively reduced DMM by 436 per 10,000 in men and 53 in women over 10 years [34]. Another study found a long-term effect for the Mexican diabetes prevention program PREVENIMSS with a reduction in DMM of 3.6 per 100,000 per year [35]. While these effects are higher than our results, this can at least be explained by the fact that ISCHG did not focus on DM prevention but could include a wide range of prevention targets and target groups, e.g. children.

Important limitations of our study may result from measurement inaccuracies of the exposure including the starting year, level of implementation, and intensity of the ISCHP-based programs. The year when a community joined a program to establish an ISCHP may only be a crude indicator for the year the community actually started the ISCHP-based interventions, which is more of a process. Interventions may have been started either years before joining the program or may not be completed until years after joining the program. The German Cooperation Network 'Equity in Health' distinguishes three levels of implementing an ISCHP [36]: sporadic informal integration, ongoing partially formalised integration, or ongoing fully formalized integration; we could not account for this characteristic (which is also changing over time) due to lacking data. Besides, our data do not include distinctions in the priorities of the various ISCHP that affect the intensity, the target groups, or the composition of the measures. For example, ISCHP focussing on programs such as No Child Left Behind (KeKiz) have certainly no or only a marginal direct effect on DMM. However, participating in an ISCHP program may reflect a fundamental, ‘beneficial to health’ attitude of a community leading to better overall health outcomes. However, these inaccuracies would underestimate the effect of ISCHP. Future evaluations should include more precise characteristics on each ISCHP associated program.

We cannot exclude a contamination of the CG. Even those communities that do not belong to a program for building an ISCHP may apply concepts and principles belonging to the core elements of an ISCHP. However, the prevention strategy in communities without an ISCHP is certainly less holistic and addresses only a subset of the social determinants of health or only a few target groups and health outcomes. Furthermore, in communities without an ISCHP, intersectoral networking and collaboration is supposed to be less intensive. In spite of these possible overlaps of IG and CG, we found positive effects in favour of the IG suggesting even higher effects of ISCHP on DMM.

Performing FE analyses, we found a notable reduction in DMM—a highly prevalent and relevant health outcome. We cannot exclude a selection bias. However, further inaccuracies to classify IG and CG tend to underestimate the ISCHP effect. In sum, our retrospective study provides at least preliminary evidence on the effectiveness of ISCHP in Germany. Further efforts should address thorough development and implementation of high-quality evaluation methods to further strengthen evidence-based integrated health promotion. Longitudinal county-level data may be an efficient data source to evaluate complex interventions.

## Supplementary Information

Below is the link to the electronic supplementary material.

Supplementary file1 (DOCX 30 KB)
